# Osteopontin-a splice variant is overexpressed in papillary thyroid carcinoma and modulates invasive behavior

**DOI:** 10.18632/oncotarget.10468

**Published:** 2016-07-07

**Authors:** Luciana Bueno Ferreira, Catarina Tavares, Ana Pestana, Catarina Leite Pereira, Catarina Eloy, Marta Teixeira Pinto, Patricia Castro, Rui Batista, Elisabete Rios, Manuel Sobrinho-Simões, Etel Rodrigues Pereira Gimba, Paula Soares

**Affiliations:** ^1^ Instituto de Investigação e Inovação em Saúde, Universidade do Porto, 4200-135 Porto, Portugal; ^2^ Institute of Molecular Pathology and Immunology of the University of Porto (Ipatimup) – Cancer Signalling and Metabolism, 4200-465 Porto, Portugal; ^3^ Medical Faculty, University of Porto, P-4200 Porto, Portugal; ^4^ Department of Pathology, Hospital de S. João, P-4200 Porto, Portugal; ^5^ INEB – Instituto de Engenharia Biomédica, 4200-135 Porto, Portugal; ^6^ ICBAS – Instituto de Ciências Biomédicas Abel Salazar da Universidade do Porto, 4050-313 Porto, Portugal; ^7^ Research Coordination, National Institute of Cancer, Rio de Janeiro 22743-051, Brazil; ^8^ Natural Sciences Department, Health and Humanities Institute, Fluminense Federal University, Rio de Janeiro 28895-532, Brazil

**Keywords:** osteopontin splice variants (OPN-SV), osteopontin-a (OPNa), thyroid cancer, migration, invasion

## Abstract

Osteopontin (OPN) is a matricellular protein overexpressed in cancer cells and modulates tumorigenesis and metastasis, including in thyroid cancer (TC). The contribution of each OPN splice variant (OPN-SV), named OPNa, OPNb and OPNc, in TC is currently unknown. This study evaluates the expression of total OPN (tOPN) and OPN-SV in TC tissues and cell lines, their correlation with clinicopathological, molecular features and their functional roles. We showed that tOPN and OPNa are overexpressed in classic papillary thyroid carcinoma (cPTC) in relation to adjacent thyroid, adenoma and follicular variant of papillary thyroid carcinoma (fvPTC) tissues. In cPTC, OPNa overexpression is associated with larger tumor size, vascular invasion, extrathyroid extension and *BRAF^V600E^* mutation. We found that TC cell lines overexpressing OPNa exhibited increased proliferation, migration, motility and *in vivo* invasion. Conditioned medium secreted from cells overexpressing OPNa induce MMP2 and MMP9 metalloproteinases activity. In summary, we described the expression pattern of OPN-SV in cPTC samples and the key role of OPNa expression on activating TC tumor progression features. Our findings highlight OPNa variant as TC biomarker, besides being a putative target for cPTC therapeutic approaches.

## INTRODUCTION

Thyroid cancer (TC) is the most common endocrine malignancy, being the fifth most frequent cancer in women [1]. TC incidence rates have been increasing in the last three decades all over the world [2]. The reason for the raised incidence of TCs remains controversial. While some studies point to improved diagnostic approaches [3, 4], others indicate that it may be correlated to environmental and lifestyle changes [5–8].

The majority of TCs are derived from follicular cells, being differentiated thyroid cancer (DTC), comprising papillary thyroid cancer (PTC) and follicular thyroid cancer (FTC), the most common subtypes and accounting for 90–95% of all cases [9]. Within PTCs, which encompass more than 80% of TCs, classical type of PTC (cPTC) corresponds to around 50% of the cases, whereas follicular variant PTC (fvPTC) corresponds to about 40% of all PTCs [10].

Despite the overall good prognosis of DTCs, a subset of DTC patients follows a more aggressive disease course, developing recurrent or metastatic disease [11]. The early identification of this cases is one of the most challenging tasks in thyroid oncology.

Osteopontin (OPN) is a secreted extracellular matrix (ECM) protein encoded by the highly conserved *SPP1* gene [12]. Previous studies have found that total OPN (tOPN) is overexpressed in TCs [13–17], similarly as reported in other tumor models, being correlated with poor survival [17]. tOPN effects in tumor progression has been associated with its ability to induce ECM invasion and migration [18]. Proteolysis and remodeling of the ECM represent early events modulating cancer cell invasion through the surrounding stroma [19, 20], and OPN has been implicated in such processes [21, 22].

OPN primary transcript is subjected to alternative splicing, generating three OPN splicing variants (OPN-SV): the full-length OPNa and the shorter variants OPNb and OPNc (lacking exons 5 and 4, respectively) [23]. Recent studies have shown that OPN-SV are differentially expressed and may exhibit functional differences in normal tissue and their respective tumors [24]. For instance [25], in hepatocellular carcinoma (HCC), tumor tissues predominantly expressed OPNa and OPNb, while normal liver tissues mainly expressed OPNc. In this tumor model, OPNa and OPNb induced Hep3B cell migration, while OPNc had no significant effect. However, in SK-Hep1 cells OPNc suppressed the migratory activity, while OPNa induced no significant changes [25]. Our group previously demonstrated that OPNc, but not OPNa and OPNb, is specifically expressed in ovarian cancer samples. Furthermore, OPNc overexpression in OvCar-3 cells activates proliferation, migration, invasion and colony formation, as well as tumor formation in nude mice [26].

Based on these data, we then hypothesized that specific OPN-SV could be putative biomarkers in TCs. We here investigated the expression patterns and putative biological roles of tOPN and OPN-SV in DTC tumor progression.

## RESULTS

### tOPN protein is overexpressed in cPTC and is associated with vascular invasion and extrathyroid extension

**Figure 1:**
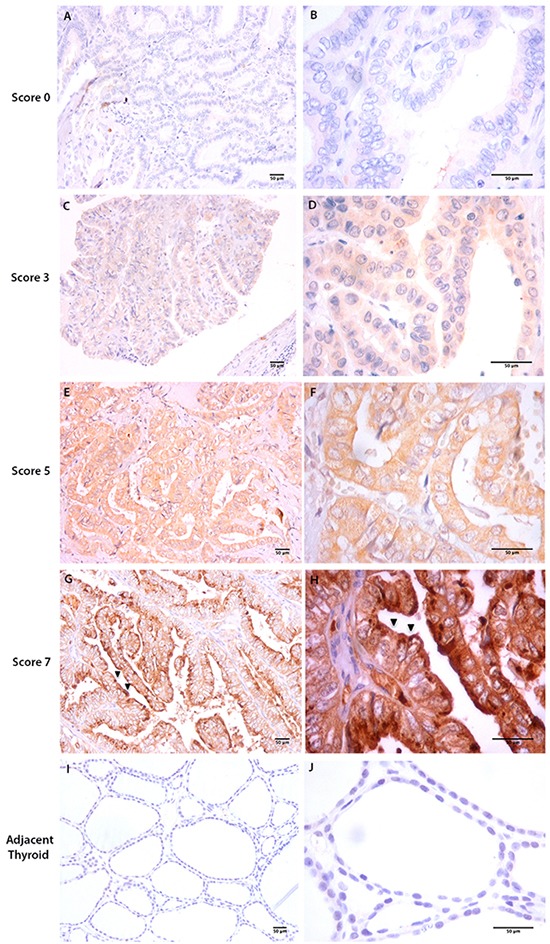
Total OPN (tOPN) IHC staining in cPTC samples Representative sections of cPTC samples showing tOPN staining in thyroid tumor cells (20x and 60x magnification, respectively at the left and right images are shown **A-H.** Scale bar: 50 μm). A and B represent cPTC samples with staining score 0; C and D, staining score 3; E and F, staining score 5; G and H staining score 7. Black arrows points to membrane tOPN staining (G and H); **I** and **J**: represent adjacent thyroid tissues negative for tOPN staining.

In thyroid tumor tissues (Figure 1A-1H), tOPN staining was mainly localized in the cytoplasm of TC cells, although a few samples also showed focal membrane staining (Figure 1G and 1H). Total OPN protein expression was observed in 27 of 44 cPTCs (61.4%), in 6 of 16 fvPTCs (37.6%) and in 6 of 10 FTCs (60%). In the cPTC, staining intensity was faint in 31.8%, moderate in 20.5% and strong in 9.1% of the cases (Table 1). Staining score of the positive cases was moderate to high in the majority of the cases (Table 1). Representative sections of tOPN staining scores from 0 to 7 are also depicted in Figure 1. Tissues adjacent to thyroid tumor areas were virtually negative for tOPN staining (Figure 1I and 1J).

**Table 1:** Staining intensity, proportion of positive stained cells and staining score of tOPN IHC in cPTC samples

Staining intensity	n	%	Proportion of positive stained cells %	n	%	OPN Staining Score*	%
**Absent**	17	38.6	**<5%**	22	50	0	38.6
						1	2.3
**Faint**	14	31.8	**5-25%**	6	13.6	2	15.9
						3	11.4
**Moderate**	9	20.5	**25-50%**	4	9.1	4	4.5
						5	13.6
**Strong**	4	9.1	**50-75%**	1	2.3	6	11.4
						7	2.3
			**75-100%**	11	25		
**Total**	44	100		44	100	44	100

* Staining intensity plus % of positive stained cells

Of note, cPTC samples displaying vascular invasion exhibited higher tOPN staining scores than tumors without vascular invasion (Table 2). Tumors displaying extrathyroid extension had higher average tOPN staining score than tumors without this feature, although not attaining statistical significance (Table 2). cPTC samples containing hyaline stroma exhibited higher tOPN staining score than tumors without stroma (p = 0.01) (Table 2). In cPTC samples, no significant associations were observed between tOPN staining scores and patient' gender or age, tumor size, capsular invasion, lymph node metastasis, thyroiditis, *RET/PTC* translocation, *BRAF^V600E^, RAS* and *TERT* mutations. Moreover, no significant associations were observed between tOPN staining score and any clinicopathological or molecular features in FTC (data not show). With regard to tOPN protein expression in well and poorly circumscribed fvPTC cases, there is a difference in the tOPN staining score media, although not attaining statistical significance (Supplementary Table S1).

**Table 2:** tOPN protein expression evaluated by IHC and correlation with clinicopathological associations in formalin-fixed paraffin-embedded (FFPE)

Tissue	Clinicopathological features (N)	tOPN tissue expression (Average ± SD)	p-value
**cPTC**	Stroma		
	Absent (n=17)	1.53 ± 2.18	**p=0.01**
	Hyaline (n=19)	3.37 ± 2.16	
	Vascular Invasion		
	Absent (n=16)	1.44 ± 2.15	**p=0.05**
	Present (n=23)	2.91 ± 2.41	
	Extrathyroid Extension		
	Absent (n=21)	0.67 ± 1.98	p=0.07
	Present (n=18)	3.00 ± 2.56	

### OPNa is the predominant OPN-SV expressed in cPTC tissues and thyroid cell lines and is associated to invasiveness

**Figure 2:**
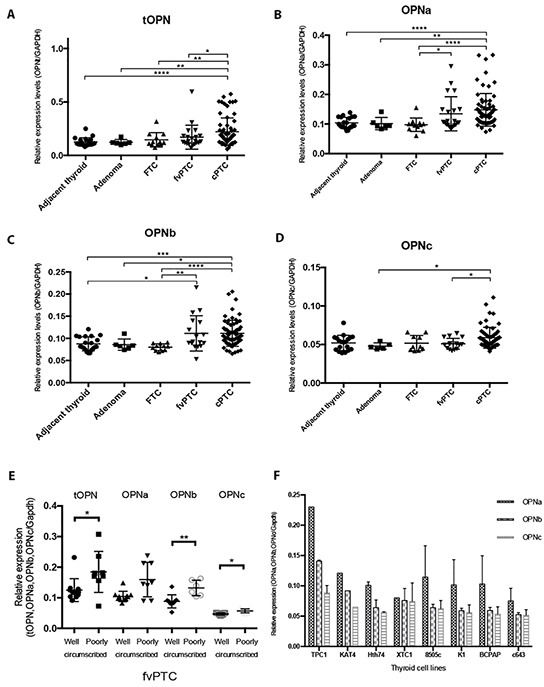
Transcript expression levels of tOPN, OPNa, OPNb and OPNc in thyroid tissue samples and in thyroid cell lines **A.** tOPN, **B.** OPNa, **C.** OPNb and **D.** OPNc mRNA expression levels has been measured by real time PCR in the distinct thyroid tissue samples (symbols: adjacent thyroid -•; follicular adenomas ■; FTC ▲; fvPTC ▼; cPTC -♦); **E.** mRNA expression levels have been measured by real time PCR in well and poorly circumscribed fvPTC tissue samples (symbols: tOPN expression in well circumscribed fvPTC -•; tOPN expression in poorly circumscribed fvPTC -■; OPNa expression in well circumscribed fvPTC -▲; OPNa expression in poorly circumscribed fvPTC -▼; OPNb expression in well circumscribed fvPTC -♦; OPNb expression in poorly circumscribed fvPTC -○; OPNc expression in well circumscribed fvPTC - •; OPNc expression in poorly circumscribed fvPTC -Δ) **F.** OPN-SV transcript expression levels have been also evaluated in distinct thyroid tumor cell lines (TPC1, KAT4, Hth74, XTC1, 8505c, K1, BCPAP and c643). * p < 0,05; ** p < 0,01; **** p < 0,0001. Results are representative of at least two independent assays with triplicates.

Since we had observed a higher tOPN protein expression in cPTC and a significant association with invasive features, we then evaluated the mRNA expression patterns of each OPN-SV (OPNa, OPNb or OPNc) and of tOPN, in an attempt to evaluate their relative contributions to cell invasiveness. The comparison of tOPN and each OPN-SV expression levels among distinct thyroid tissue samples showed that tOPN is overexpressed in relation to each OPN-SV (Supplementary Figure S1). It is worth to mention, that tOPN expression corresponds to the sum of all OPN-SV. Among the three OPN-SV, OPNa has the highest expression levels in all thyroid tissues (Figure 2A and Supplementary Figure S1).

cPTC samples express higher levels of tOPN, OPNa and OPNb, when compared to adjacent thyroid, adenoma and FTC samples (p < 0.05) (Figure 2A; 2B and 2C). OPNc variant has the lowest expression levels and is only significantly overexpressed in cPTC samples in relation to adenoma and fvPTC samples (Figure 2D). These data show that OPNa splice variant is overexpressed in cPTCs when compared to other thyroid tissues. In an attempt to understand the expression pattern of OPN-SV in fvPTC cases, we separately analyzed well or poorly circumscribed fvPTC cases. We observed that poorly circumscribed fvPTC significantly overexpress tOPN, OPNb and OPNc variants, compared with well-circumscribed cases of fvPTC (p = 0.02, p = 0.002 and p = 0.01; respectively). Also a higher expression of OPNa in poorly circumscribed fvPTC was noted, although not reaching statistical significance (p = 0.07) (Figure 2E). The median transcript expression levels of tOPN were significantly higher in cPTC tumors larger than 2 cm (Table 3). tOPN and OPNa median expression levels were significantly higher in cPTC samples with extrathyroid extension and vascular invasion than in those without such features (Table 3). cPTC harboring *BRAF^V600E^* gene mutation exhibited higher tOPN, OPNa and OPNb transcript expression levels than those presenting wild type *BRAF gene* (p < 0.03) (Table 3). In fvPTC, older patients presented higher tOPN and OPNa expression levels (p < 0.04). No statistical significant differences have been observed between tOPN and OPN-SV transcript expression levels and other clinicopathological or molecular features in fvPTC.

**Table 3:** Correlation between tOPN and OPN-SV transcript expression levels with clinicopathological and molecular features in cPTC and fvPTC samples

Tissue	Variable	tOPN mRNA expression (Median)	p-value	OPNa mRNA expression (Median)	p-value	OPNb mRNA expression (Median)	p-value
**cPTC**	Tumor size (cm)						
	<2 (n=18)	0.14	**p=0.02**	0.12	p=0.27	0.10	p=0.89
	≥ 2 (n=38)	0.17		0.13		0.10	
	Extrathyroid Extension						
	Absent (n=20)	0.15	**p=0.04**	0.12	**p=0.02**	0.09	p=0.09
	Present (n=21)	0.18		0.13		0.11	
	Vascular Invasion						
	Absent (n=22)	0.15	**p=0.04**	0.12	**p=0.03**	0.10	p=0.45
	Present (n=28)	0.17		0.13		0.10	
	*BRAF*^V600E^ Mutation						
	Absent (n=26)	0.14	**p=0.03**	0.12	**p=0.01**	0.09	**p=0.01**
	Present (n=30)	0.18		0.13		0.11	
**fvPTC**	Age (yr)						
	<45 (n=13)	0.11	**p=0.03**	0.09	**p=0.04**	0.08	p=0.07
	≥45 (n=9)	0.17		0.14		0.11	

Regarding OPN-SV expression patterns in TC cell lines, we found that OPNa variant is also overexpressed when compared to OPNb and OPNc variants in all the tested TC cell lines, except XTC1 (Figure 2F).

### OPNa overexpression modulates proliferation and migration in c643 and 8505c cell lines

**Figure 3:**
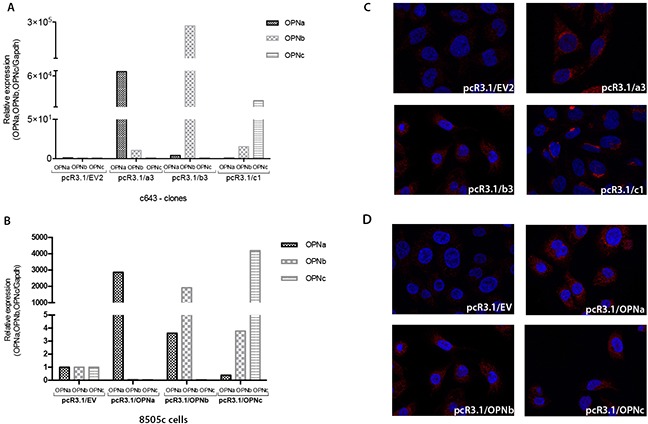
Stable overexpression of each OPN-SV in c643 and 8505c transfected cells The expression levels of each OPN-SV were analyzed by qRT-PCR, when compared to control cells transfected with EV plasmid, which was used as the reference sample. The relative expression levels of each OPN-SV were calculated using the delta-delta CT method (pcR3.1/OPNa, pcR3.1/OPNb or pcR3.1/OPNc relative to pcR3.1/EV). Each OPN-SV is represented by a different bar graph, as indicated. **A.** OPN-SV mRNA overexpression in c643 cells; **B.** OPN-SV mRNA overexpression in 8505c cells. **C.** Immunocytochemistry analyses of OPN expression in control cells (c643 cells with EV: pcR3.1/EV) and in c643 cells overexpressing OPNa (pcR3.1/OPNa), OPNb (pcR3.1/OPNb) and OPNc (pcR3.1/OPNc) have been performed using the anti-tOPN antibody; **D.** Immunocytochemistry analyses of OPN expression in control cells (8505c cells with EV: pcR3.1/EV) and in 8505c cells overexpressing OPNa (pcR3.1/OPNa), OPNb (pcR3.1/OPNb) and OPNc (pcR3.1/OPNc). NOTE: Cell isolated clones used for these assays were named EV2, a3, b3 and c1 for each OPN-SV.

c643 and 8505c cells transfected with each OPN-SV express higher transcript levels of the corresponding ectopically expressed OPN-SV in relation to EV control cells (Figure 3A and 3B) and were used for further functional assays, as depicted below. The overexpression of OPN protein in c643 and 8505c cell lines transfected with tOPN and each OPN-SV was validated using immunocytochemistry (Figure 3C and 3D).

**Figure 4:**
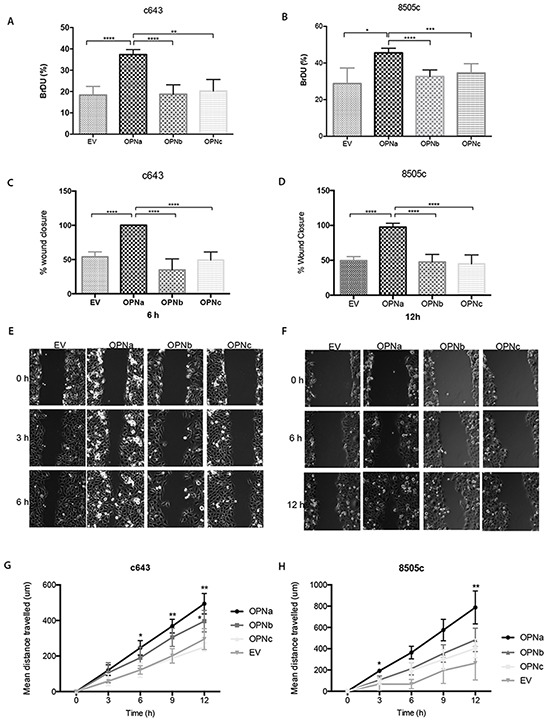
Cells overexpressing OPNa stimulate cell growth, migration and motility DNA synthesis and proliferation rates have been evaluated by BrdU incorporation assays; **A.** c643 and **B.** 8505c cell clones (overexpressing EV, OPNa, OPNb, OPNc, respectively). Effect of each OPN-SV overexpression in c643 **C.** and in 8505c **D.** cells on migration rates using wound-healing assays. Representative images are shown for cell migration in c643 **E.** and 8505c **F.** OPN-SV overexpressing cells, which were monitored by time-lapse microscopy for 0, 3, 6, 9 and 12 hours after cell scratch. The % wound closure in the graphs represents the wound measured area. For motility assays, OPNa, OPNb, OPNc and EV cell clones were cultured in μ-Slide 4 Well ^Ph+^ and monitored by time-lapse microscopy for 12 hours. Four microscope fields were averaged for each c643 **G.** and of 8505c **H.** OPN-SV overexpression clones and controls containing 10 cells/field (Representative videos are available in Online Supplemental Material). Graph data corresponds to motility rates from two independent experiments and values are expressed as mean ± se. *p < 0,05; ** p < 0,01; **** p < 0,0001. All the experiments were done in triplicated.

c643 and 8505c OPNa overexpressing cells displayed higher proliferation rates than OPNb, OPNc and EV control at 48 h (p < 0.01) (Figure 4A, 4B; p < 0.05).

In order to investigate the effect of OPN-SV overexpression on c643 and 8505c cell migration, these cells were subjected to *in vitro* wound closure assays (Fig. 4C, 4D, 4E and 4F). c643 clones overexpressing OPNa have higher migration rates, as depicted by wounding area, than OPNb, OPNc or EV clones. At 6h after cell scratch, c643-OPNa overexpressing cells completely closed the wound edges, at variance with OPNb, OPNc or EV clones. The same migration behavior was observed for 8505c-OPNa overexpressing cells, although the wound closure has only been achieved at 12h after cell scratch. In order to further validate these motility properties, we used time-lapse video microscopy, monitoring the distance travelled by the cells during 12h. As shown in Figure 4G, c643-OPNa overexpressing clones showed higher motility rates than the remaining OPN-SV clones and EV controls. Similar higher motility behavior pattern has been observed for 8505c-OPNa overexpressing cells (Figure 4H) (Representative videos are shown in Supplemental Material).

### TC cells overexpressing OPNa induces MMP2 and MMP9 activity

**Figure 5:**
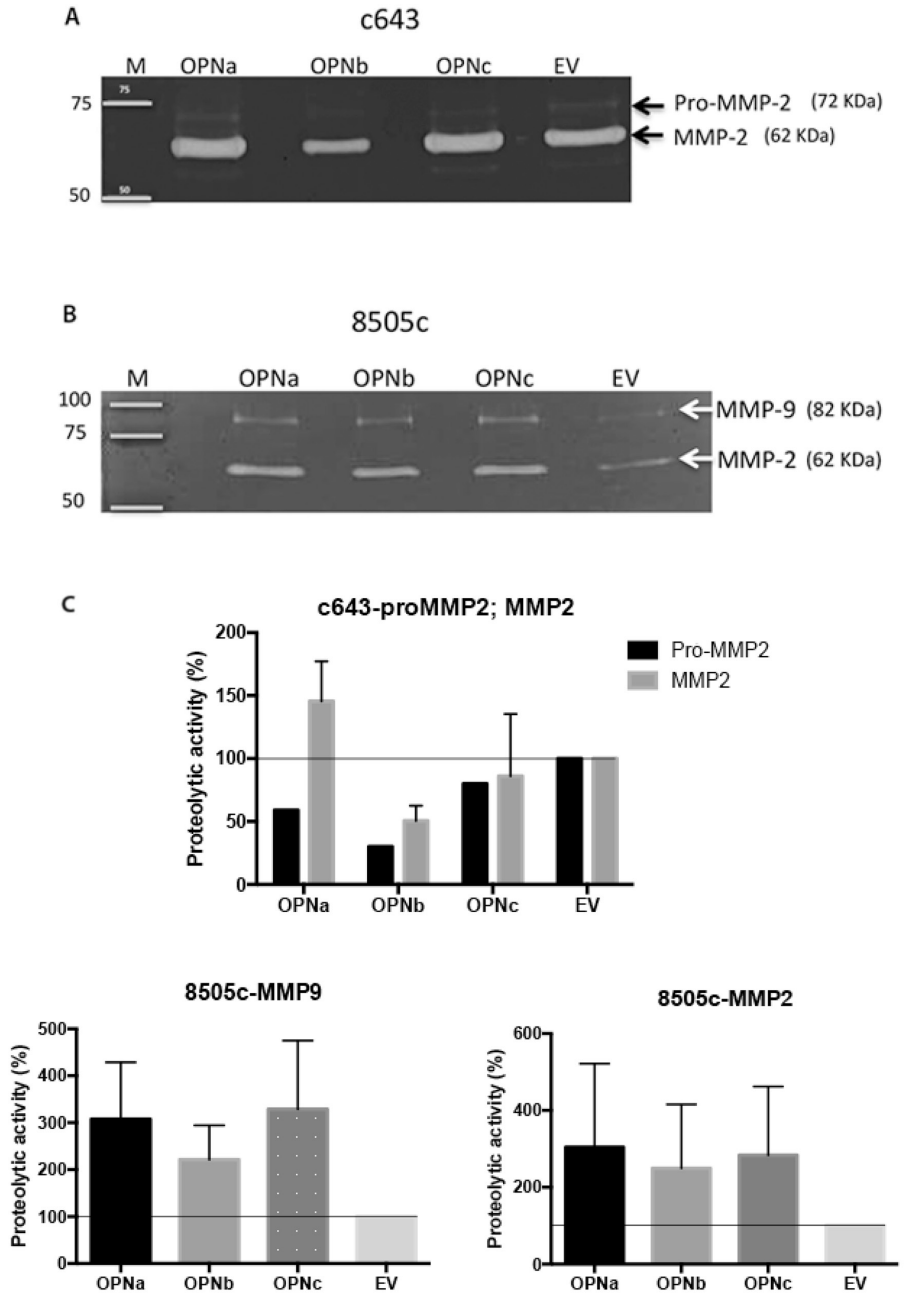
MMP2 and MMP9 activity in cells overexpressing OPN-SV Gelatin zymography assay was used to analyze the levels of active MMP2 and MMP9 in conditioned medium from **A.** c643 (overexpressing OPNa, OPNb, OPNc and EV) and from **B.** 8505c (overexpressing OPNa, OPNb, OPNc and EV) cell clones. Representative images are shown. On the left are depicted molecular weights of standard markers; on the right are shown the variants of metalloproteinases (MMPs) observed in the gels. **C.** The intensity of gelatin-digested bands by pro- MMP2, MMP2 and MMP9 were measured by densitometry and are represented by the diagram bar. Percentage (%) of proteolytic activity from 8505c and c643 cells overexpressing OPNa, OPNb or OPNc was compared with the activity present in culture medium from 8505c and c643 control cells (EV). Data correspond to mean values of two independent experiments.

We then investigated the impact of OPNa overexpression in invasion-related enzymes secreted at the extracellular conditioned medium (CM). CM secreted from c643 and 8505c cells overexpressing OPNa, OPNb, OPNc or EV control were tested for MMP2 and MMP9 metalloproteinase activity. We found that the levels of matrix MMP2, mainly the active form, were increased in the CM secreted from c643-OPNa overexpressing cells, compared to CM secreted from the corresponding c643-OPNb and c643-OPNc clones (Figure 5A). No MMP9 expression was detected in c643 cells, regardless of the expressed OPN-SV. We observed an increase in the activity of MMP2 and MMP9 in the CM secreted from 8505c-OPNa overexpressing cells (Figure 5B). 8505c-OPNa overexpressing cells present higher levels of active MMP2 and MMP9 than 8505c-OPNb and 8505c-OPNc overexpressing cells.

### Overexpression of OPNa increases the invasive potential of TC cells in the CAM assay

*In vivo* CAM assays were conducted to evaluate the effect of ectopic OPNa overexpression on the angiogenic, tumorigenic and invasive behavior of c643 cells. To achieve this, the c643 clones overexpressing OPNa and EV were injected in the chick embryo CAM.

**Figure 6:**
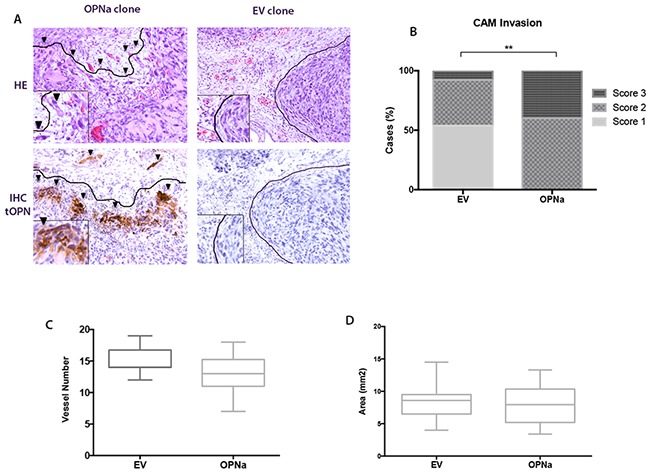
Overexpression of OPNa increases the invasive potential of TC cells in the CAM assay **A.** Invasive capacity of inoculated cells was evaluated by HE and IHC using an anti-tOPN antibody in xenograft CAM tumor sections. Positive human tOPN immunostaining demonstrating effective overexpression of OPN in CAM tumors originated from OPNa clones. c643-EV cells forming compacted tumors (score 1; full lines surrounding the tumor bulk, right panels). OPNa tumors exhibiting loosen structures and single cells invading the xenograft CAM tumor mesenchyme (arrow heads; score 3, left panels); **B.** Invasion score analysis demonstrating c643-OPNa overexpressing cells and their corresponding invasive score, when compared to EV control clones (p = 0.003) **C.** CAM angiogenic assay, as demonstrated by the number of formed vessels in c643-OPNa and EV control clones **D.** CAM xenograft tumors areas measured in mm^2^ indicating tumorigenesis in c643-OPNa and EV clones.

Tumors formed by cells overexpressing OPNa exhibited an invasive pattern (Figure 6A, left). In contrast, xenograft tumors formed by c643-EV cells were compact, with encapsulated-like borders (Figure 6A, right), as evaluated by HE staining (Figure 6A, upper panel) and IHC for tOPN staining (Figure 6A, lower panel). We used a score system to semi-quantify the property of TC cells to spread inside the CAM. We observed that c643 cells overexpressing OPNa exhibited higher invasive capacity (p = 0.003) than c643-EV cells. This higher invasiveness was characterized by the presence of tumor cells oriented towards the invasion front and the presence of isolated cells and small clusters at distance from the tumor bulk (Figure 6B). Similar angiogenic (p > 0.05; Figure 6C) and tumorigenic responses (p > 0.05; Figure 6D) were observed in c643-OPNa when compared with c643-EV control.

## DISCUSSION

We characterize for the first time the transcript and protein expression patterns of tOPN and OPN-SV in DTC and TC cells. Then, we also showed their association to TC prognosis and progression features, especially in cPTC samples. Our data further demonstrated that OPNa transcript is the dominant overexpressed splice variant in DTC tissues and in distinct thyroid cell lines, notably in cPTC. Remarkably, high transcript expression levels of tOPN and OPNa, but not OPNb and OPNc, were associated with aggressive cPTC clinicopathological features (tumor size, vascular and extrathyroid invasion). Moreover, we demonstrated using *in vitro* and *in vivo* approaches that ectopically overexpressed OPNa promotes cell growth, migration and invasion in TC-derived cell lines.

We have shown that tOPN is predominantly expressed (both at transcript and protein level) in cPTC and in fvPTC, when compared to FTC and adjacent non-tumoral thyroid tissues. In fvPTC samples, we observed higher expression of tOPN, OPNa and OPNb variant in poorly circumscribed cases. This result is very interesting for several reasons: a) first, it is in accordance with the increased expression of OPN and the respective spliced forms in cases presenting invasive features; b) second, it corroborates our *in vitro* and *in vivo* results showing an increased invasiveness in cells overexpressing tOPN and OPN-SV; c) finally, it shows the different biological characteristics of the well and poorly circumscribed fvPTC. In this last point it is also worth to mention that our results in OPN expression fits with the recent proposal for the reclassification of encapsulated fvPTC as NIFT (“noninvasive follicular thyroid neoplasm with papillary-like nuclear features”) due to the very low risk of adverse outcome of these patients [27].

Further, we found that high levels of tOPN expression in cPTC are associated with increased tumor size, presence of extrathyroid extension, vascular invasion and *BRAF^V600E^* mutation. Our results regarding tOPN are in accordance with previous studies demonstrating tOPN overexpression in PTC samples [15]. Our results also corroborates previous studies which showed that tOPN overexpression (transcript and/or protein) are significantly associated with poor prognostic factors, such as presence of lymph node metastasis, tumor size and poor disease free survival in PTC samples [13, 14, 16, 17].

We also observed that tOPN protein overexpression in cPTC is significantly associated with hyaline stroma. These data are in accordance with OPN as a glycoprotein secreted in the extracellular matrix, both in tumor and non-tumoral tissues [28]. OPN is also known to be upregulated in the tissue stroma in different conditions, such as salivary pleomorphic adenomas [29] and intrahepatic cholangiocarcinoma [30]. It is well known that the interplay between epithelial cells and the microenvironment may contribute to keep the epithelial polarity and to modulate growth inhibition [31]. On the other hand, the stromal compartments undergo changes in response to emerging epithelial lesions that can have a key role in cancer initiation and progression [31, 32]. Accordingly, our observed data regarding the correlation of tOPN and the presence of a hyaline stroma support the assumption that either tOPN expression alone or tOPN in association to hyaline stroma may play a role in cPTC tumor aggressiveness.

Our data show that OPNa variant is overexpressed and specifically associated with poor prognostic features in PTC. In particular, OPNa variant was significantly associated with presence of extrathyroid extension, vascular invasion and *BRAF^V600E^* mutation. In contrast to this, OPNb was only significantly associated with *BRAF^V600E^* mutation. In the fvPTC, the only significant association observed was between high tOPN and OPNa expression levels and older patients. Based on these findings, we postulate that elevated OPNa expression in TC may occur during tumor progression, facilitating more aggressive phenotypes. Some authors have shown that OPN-SV expression and its associations to clinicopathological features seem to be tissue specific. For instance, it has been reported that high OPNb and OPNc expression levels in breast cancer [33] and in gastric tumors [34] is correlated with more aggressive clinicopathological features. In a combined expression analysis, OPNc, ER and HER2 can reliably predict grade 2-3 in breast cancer samples [35]. Moreover, in a prostate tumor model, our group has also observed that OPNc overexpression is correlated to poor prognostic features [36]. Conversely, our data show that OPNa has a more relevant prognostic role in PTC than the other OPN-SV.

To assess the possible impact of OPN-SV in TC cell properties we ectopically overexpressed OPNa, OPNb or OPNc in c643 and 8505c cell lines with plasmid constructs containing each of the three OPN-SV. We observed that TC cells overexpressing OPNa displayed significantly increased cell growth, migration and motility, whereas OPNb and OPNc overexpression in TC cells did not induce similar effects. Other reports have demonstrated that OPN-SV play an important role in tumor progression by regulating cell growth, adhesion, migration and tumor formation. As reported by Lin J and co-workers [37], OPNb overexpressing cells derived from esophageal adenocarcinoma also evoked enhanced cell proliferation, migration and invasion. Additionally, our group also previously demonstrated that OPNc activates invasion and adhesion properties, as well as metastatic potential and angiogenesis in a prostate and an ovarian carcinoma model [26, 38]. In prostate cancer cells, OPNc can activate AR signaling [39] and resistance to docetaxel [40]. Furthermore, other groups demonstrated that in hepatocellular carcinoma cells, OPNa and OPNb can induce cell migration [25]. Our findings highlight the importance of OPNa in TC cell growth and migratory and invasive phenotype.

To further explore OPNa roles on modulating TC invasive properties, we also explored the role of OPNa in the activation of matrix metalloproteinases (MMPs). MMPs are important enzymes in the metastatic cell arsenal. These proteins can degrade both cell adhesion molecules and extracellular matrix molecules, enabling tumor cells both to migrate from the tumor bulk and to invade adjacent tissues [41]. Previous data from our group in prostate and ovarian tumor models showed that cells overexpressing the OPNc variant induce expression of MMP2 and MMP9, highlighting the functional tissue specificity of OPN-SV [38]. Thyroid carcinomas produce elevated levels of MMP2, which has been correlated with the presence of lymph node metastasis [51]. Herein, we have found that CM collected from cells overexpressing OPNa have increased activity of MMP2 in c643 cells and MMP2 and MMP9 in 8505c cell lines. These results evidence that OPNa may promote TC cell invasion through inducing MMP2 and MMP9 secretion. The detailed mechanism by which this event occurs still needs further characterization. Nonetheless, it has been described that OPN can regulate MMPs activity by binding to pro-MMP9, thus promoting its activation [42]. Additionally, OPN can induce NFkB-mediated pro-MMP2 and MMP9 activation through IkBa/IKK signaling pathway [43].

Since MMPs expression and cancer cell migration are fundamental features for tumor invasion [44], we further investigated the contribution of OPNa variant for a TC cell line invasiveness using an *in vivo* experimental model. Using the CAM assay approach, we observed that tumors formed by these cell clones present a loosen structure, in which the cells were oriented towards the invasion front while single cells and cell clusters invading the CAM mesenchyme could also be observed. In contrast to this, EV clones formed compact tumors, with clear defined boarders lacking invading cells. Similarly to our data, overexpression of OPNa has been previously associated with the cancer cell invasion modulation in mesothelioma, breast cancer and hepatocellular carcinomas [25, 45, 46]. Although activating cell invasion, OPNa overexpression does not significantly modulated angiogenesis and tumorigenesis in this *in vivo* tumor model. Some oncogenic proteins can activate some specific steps in tumor progression, but not others. As evidenced by our data OPNa overexpression *in vivo* may predominantly modulate signaling pathways that stimulate migration and invasion, possibly through stimulating extracellular matrix degradation by MMP2 and MMP9. In light of our results, we may hypothesize that the increased invasive capacity of c643 cells overexpressing OPNa (in comparison to c643-EV cells) allows the cells to reach the blood vessels, and have no effect in the recruitment of new vessels.

In conclusion, our data demonstrated that OPNa is the prevalent OPN-SV in DTC tissues and cell lines, and that overexpression of OPNa is associated with poor prognostic and invasive features in cPTC. Moreover, OPNa overexpression in TC cell lines strongly increases cell migration, invasion and MMPs activity, evidencing a major role for OPNa in TC progression features. Taken together, these features provide early evidence that OPNa can potentially mediate invasive and metastatic potential in cPTCs.

## MATERIALS AND METHODS

### Tumor specimens

We evaluated adjacent thyroid tissues (n = 20), thyroid adenomas (n = 6), follicular thyroid carcinoma (FTC) (n = 12), follicular variant of papillary thyroid carcinoma (fvPTC) (n = 22) and classic papillary thyroid carcinoma (cPTC) (n = 69). All the analyzed specimens were collected from primary tumors, surgically resected at the Centro Hospitalar São João, Porto, Portugal. After surgery, samples were immediately snap-frozen and stored at −80°C until use. Additional fragments were fixed in 10% buffered formalin and embedded in paraffin (FFPE). The histologic diagnosis of all cases were reviewed by three thyroid pathologists (CE, ER, MSS) according to the WHO classification [47]. Clinicopathological and molecular features are summarized in Supplementary Table S2. All the procedures described in this study were approved by the respective ethical boards and are in accordance with national and institutional standards.

### Immunohistochemistry

OPN IHC analysis was performed in representative tumor tissue sections of 44 cPTC, 16 fvPTC and 10 FTC samples using an antibody that recognize all three OPN-SV (anti-total OPN- tOPN) (polyclonal, goat, 1:500, R&D Systems). Normal gallbladder was used as a positive control, once it has been previously reported to overexpress tOPN [48, 49]. IHC procedure was done according to [49]. Semi-quantitative IHC evaluation was independently performed by two observers (CE and LBF). Total OPN staining was scored in the range 0-7, which corresponds to the sum of the staining intensity (absent = 0, faint = 1, moderate = 2 and strong = 3) plus the proportion of positively stained cells (<5% = 0; 5-25% = 1; 25–50% = 2, 50–75% = 3 and >75% = 4) (Table 1).

### RNA extraction, reverse transcription and real time PCR

Total RNA was extracted from cell lines and tumor tissues using Trizol reagent (Life Technologies, GIBCO BRL). For cDNA preparation, 1 μg of total RNA was reverse transcribed using the RevertAid first-strand cDNA synthesis kit (Fermentas, Burlington, ON, Canada).

Each OPN-SV transcript region was amplified with specific oligonucleotide pairs (Supplementary Table S3 and Supplementary Figure S1A). Quantitative PCR reactions were conducted using SYBR Green detection system (Applied Biosystems, Warrington WA1 4SR, UK). Conditions for OPN-SV amplification were 50°C for 2 minutes, 94°C for 5 minutes followed by 40 cycles of 94°C for 30 seconds, 60°C for 30 seconds, and 72°C for 45 seconds. Relative gene expression was calculated using the Delta-Delta CT method. GAPDH gene was used as the constitutive control.

### Cell culture, OPN plasmids and transfections

We analyzed eight TC cell lines: TPC1, KAT4, Hth74, XTC1, 8505c, K1, BCPAP and c643, from which two were selected for stable transfection. All cell lines were authenticated using DNA profile analysis, obtained with the PowerPlex 16 system (Promega, Madison, USA), according to ATCC and HSRRB available DNA profiles [50]. All the cell lines were cultured in standard culture medium, supplemented with 10% fetal bovine serum (FBS), 100 IU/ml penicillin and 100 mg/ml streptomycin in a humidified environment containing 5% CO_2_ at 37°C. The open reading frame of OPN-SV, was cloned into pCR3.1 mammalian expression vector, as previously described [51]. OPN-SV expression vectors (kindly provided by Dr. George Weber (Cincinnati University)) were used to transfect c643 and 8505c cell lines (the ones with lower OPN expression; Figure 2F) in order to overexpress each OPN-SV. These cells were also transfected with the pCR3.1 control empty vector (EV). Transfections were performed using LipofectamineTM 2000 (Invitrogen, CA). Expression plasmids were transfected into c643 and 8505c cells and the stably overexpressing cell clones were selected using 600 μg/ml of G418 for c643 and 800 μg/ml for 8505c cell lines.

### Immunocytochemistry

Cells plated on coverslips were fixed in 4% paraformaldehyde for 20 min at room temperature (RT). Cells were emerged in NH_4_Cl 50 mM in PBS during 10 min, and then, cells were permeabilized in 0.2% Triton X-100 and blocked in 5% BSA in PBS for 30 min at RT. Primary antibodies were diluted in PBS containing 5% BSA and incubated overnight at 4°C as follows: rabbit polyclonal against OPN (Rockland, Limerick, PA, USA, diluted 1:500). Coverslips were washed in 0.1% Triton X-100 prepared in PBS (PBT) and incubated with goat anti-rabbit IgG secondary antibodies conjugated with Alexa Fluor 594 (Invitrogen; diluted 1:300 in 5% BSA–PBT) for 1h at RT. Nuclei were stained with 0.1 mg/ml diamino phenylindole (DAPI; Sigma–Aldrich). Images were taken by a Zeiss fluorescence microscope with ApoTome attachment (Axio Imager Z1 stand).

### Cell proliferation

Proliferation was measured by evaluating bromodeoxy uridine (BrdU) incorporation in c643 and 8505c OPN-SV transfected cells as previously described [52]. Quantification of BrdU positive cells was performed using ImageJ software, by counting the percentage of BrdU-positive nuclei on a total of 1500 cells.

### Cell migration and motility assays

For cell migration assays, c643 and 8505c (overexpressing OPNa, OPNb, OPNc or EV) clones were seeded until forming a confluent monolayer. The cell wound was created by scraping the cell monolayer with a pipette tip. After scratch, cell migration was monitored by time-lapse microscopy and images were taken every 10 minutes for 24 hours.

For motility assays, 3,0 × 10^5^ cells of c643 and 8505c OPNa, OPNb, OPNc or EV transfected cells were plated in μ-Slide 4 Well Ph+ and monitored by time-lapse video microscopy to evaluate cell motility. Migratory tracks were measured for individual cells overexpressing each OPN-SV or EV cells using ImageJ software. Four microscope fields containing around 10 cells per field were monitored for each cell line.

### Gelatin zymography

2.5 × 10^5^ cells from c643 and 8505c (overexpressing OPNa, OPNb, OPNc or EV) were seeded to generate conditioned medium (CM). Matrix metalloproteases (MMPs), namely MMP2 and MMP9, activity was assessed in the CM of each condition by gelatin zymography, as previously described [53].

### *In vivo* chicken embryo chorioallantoic membrane (CAM) angiogenesis, tumorigenesis and invasion assays

*In vivo* angiogenic activity of c643 cells overexpressing OPNa and the EV controls were assessed by chicken chorioallantoic membrane (CAM) assay, as previously described [54]. According to the European Directive 2010/63/EU, ethical approval is not required for experiments using embryonic chicken. Correspondingly, the Portuguese law on animal welfare does not restrict the use of chicken eggs.

CAMs bearing the tumors were fixed in 10% neutral-buffered formalin and paraffin-embedded for slide sections. Sections were HE stained for histological examination or processed for anti-tOPN IHC analysis to validate OPN overexpression in CAM-xenografted tumor cells. These xenograft tissue sections were also used to evaluate cell invasion. The analysis was performed in a blind fashion manner by two independent observers and slides were scored as follows: score 1- Tumor cells are tight together forming a compact mass. The invasion front (area where tumor cells touch the CAM mesenchyme) is clearly defined as an encapsulated -like structure; score 2- Tumor cells are more loosen at the core of the tumor and in some cases, matrigel can be detected. Cells are oriented towards the invasion front; score 3- Tumor cells are oriented towards the invasion front and it is possible to observe single cells or small clusters of cells disconnected from the invasive front.

### Data and statistical analysis

Statistical analyses were performed using 22.0 SPSS statistical package (IBM, 2014). *X*2 and independent samples t-test were performed to verify if there was any association(s) between OPN expression and clinicopathological data. GraphPad was used for the construction of the graphs. ANOVA test was used to calculate significance in the CAM angiogenic and tumorigenic assays. A ChiSquare test was used to calculate significance in the CAM invasion assay. All results are presented as mean ± standard error. Values of p ≤ 0.05 were considered to be statistically significant.

## SUPPLEMENTARY FIGURES TABLES AND VIDEOS






