# Supplementary material for: Osteopontin-a splice variant is overexpressed in papillary thyroid carcinoma and modulates invasive behavior
Source: Oncotarget. 2016 Jul 7;7(32):52003–16. doi: 10.18632/oncotarget.10468 (PMC5239531; doi:10.18632/oncotarget.10468)
Supplement: Supplementary file 1 [file oncotarget-07-52003-s001.pdf]

Osteopontin-a splice variant is overexpressed in papillary thyroid carcinoma and modulates invasive behavior

SUPPLEMENTARY FIGURE, TABLES AND VIDEOS

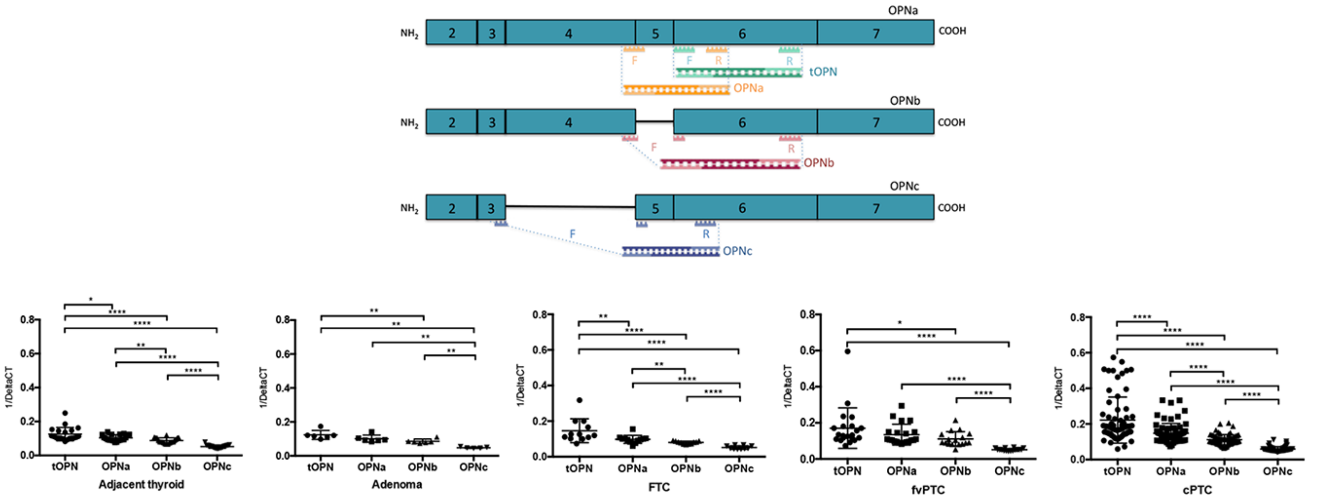

Supplementary Figure S1: Schematic representation of three OPN splice variants (OPNa, OPNb and OPNc). Figure depicts oligonucleotide primers annealing sites for amplification of each specific OPN splice variant and tOPN. OPNa (full-length splice variant), OPNb (lacks exon 5) and OPNc (lacks exon 4). Green: primers used for tOPN amplification (forward and reverse primers, located at exon 6) and the corresponding green tOPN amplification product; Orange: primers used for OPNa amplification (forward primer located at exon 4-5 splice junction; reverse primer located at exon 6) and the corresponding OPNa orange amplification product; Red: primers used for OPNb amplification (forward primer, located at exon 4-6 splice junction; reverse primer, located at exon 6) and the corresponding red OPNb amplification product; Blue: primers used for OPNc amplification (forward primer – exon 3-5 junction; reverse primer - exon 6) and the corresponding blue OPNc amplification product. Numbers in the boxes represent the coding exons. **B.** OPN-SV transcript expression levels (symbols: tOPN -•; OPNa -■; OPNb -▲, OPNc -▼) in adjacent thyroid, follicular adenomas, FTC, fvPTC and cPTC samples.

Supplementary Table S1: Analyses of total OPN (tOPN) IHC staining in well and poorly circumscribed fvPTC samples

fvPTC	N	Score Mean	
Well circumscribed	13 (81.25%)	1	
Poorly circumscribed	3 (18.75 %)	2.33	*p=0.27*
Total	16 (100%)		

Supplementary Table S2: Summary of the clinical, pathological and molecular data of the FTC, fvPTC and cPTC cases

Variable	FTC	fvPTC	cPTC
**Gender**			
**Female**	4 (66.7%)	20 (90.1%)	54 (84.4%)
**Male**	2 (33.3%)	2 (9%)	10 (15.6%)
**Age (yr) (mean ±S.D.)**	54.7 (±15.8)	40.1 (±13.1)	41.5 (±15.4)
**Stroma**			
**Absent**	-	-	17 (47.2%)
**Present**	-	-	19 (52.8%)
**Tumor size (cm)**			
**(mean ±S.D.)**	4.1 (±1.2)	2.6 (±1.5)	2.6 (±1.4)
**Extrathyroid Extension**			
**Absent**	-	10 (83.3%)	24 (51%)
**Present**	-	2 (16.7%)	23 (49%)
**Invasion (vascular and/or capsular)**			
**Absent**	0	13 (68.4%)	24 (42.8%)
**Present**	6 (100%)	6 (31.6%)	32 (57.2%)
**Lymph Node Metastasis**			
**Absent**	-	12 (75%)	25 (53.2%)
**Present**	-	4 (25%)	22 (46.8%)
**Thyroiditis**			
**Absent**	-	-	2 (20%)
**Present**	-	-	8 (80%)
***RET/PTC1* translocation**			
**Absent**	11 (100%)	21 (95.5%)	45 (83.3%)
**Present**	0	1 (4.5%)	9 (16.7%)
***BRAF*^{V600E} mutation**			
**Absent (n=26)**	11 (100%)	20 (91%)	25 (43.1%)
**Present (n=30)**	0	2 (9%)	33 (56.9%)
***TERT* mutation**			
**Absent**	7 (87.5%)	22 (100%)	51 (96.2%)
**Present**	1 (12.5%)	0	2 (3.8%)
***RAS* mutation**			
**Absent**	11 (91.7%)	18 (81.8%)	50 (92.6%)
**Present**	1 (8.3%)	4 (18.2%)	4 (7.4%)

Supplementary Table S3: Forward and reverse oligonucleotide sequences used for tOPN, OPN-SV and GAPDH specific amplification

Gene	Oligonucleotide Name	Sequence 5' – 3'
**tOPN**	tOPNF	CCA ACG AAA GCC ATG ACC AC
	tOPNR	CTG TGG GGA CAA CTG GAG TG
**OPNa**	OPNaF	ATC TCC TAG CCC CAC AGA AT
	OPNaR	CAT CAG ACT GGT GAG AAT CAT C
**OPNb**	OPNbF	CTC CTA GCC CCA CAG ACC CT
	OPNbR	TAT CAC CTC GGC CAT CAT ATG
**OPNc**	OPNcF	CTG AGG AAA AGC AGA ATG
	OPNcR	AAT GGA GTC CTG GCT GT
**GAPDH**	GAPDH-F	TGA CCC CTT CAT TGA CCT CA
	GAPDH-R	AGT CCT TCC ACG ATA CCA AA

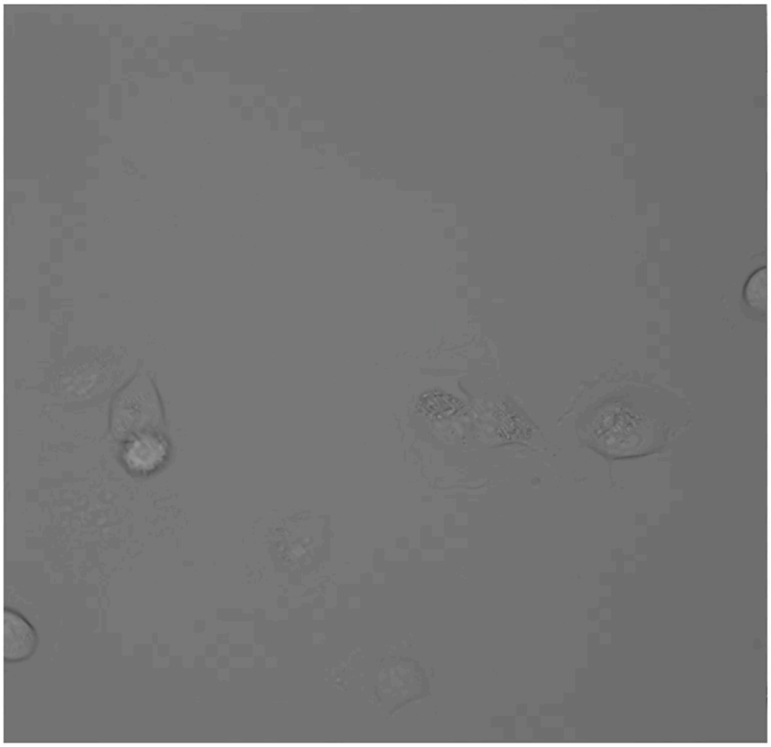

Supplementary Video S1: Motility 8505c-OPNa: Representative video of motility assay used to evaluate the motility of 8505c cells overexpressing OPNa splice variant.

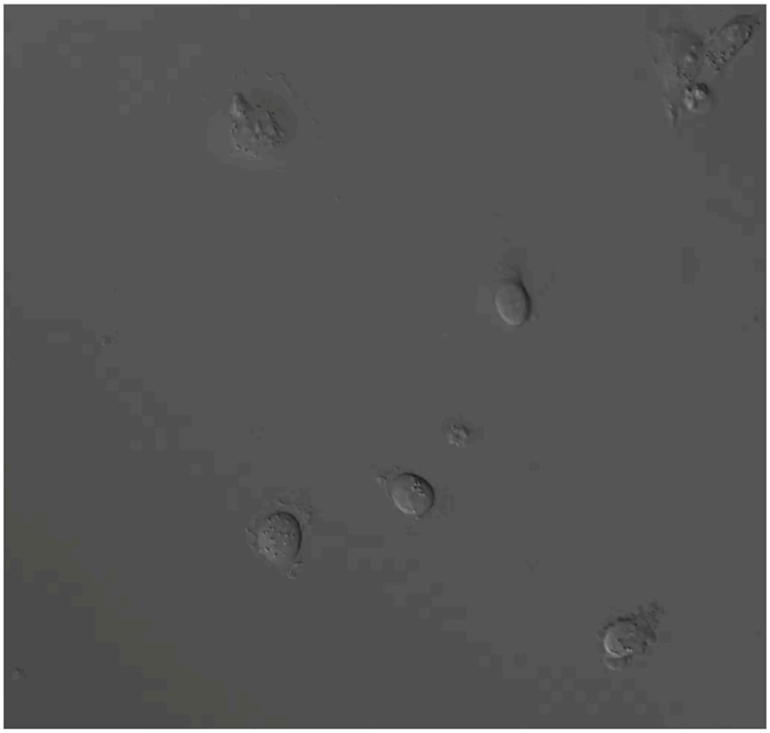

Supplementary Video S2: Motility 8505c-EV: Representative video of motility assay used to evaluate the motility of 8505c cells overexpressing empty vector (EV) control.
